# Machine learning model for predicting neuropathic pain following thoracic oncology surgery

**DOI:** 10.3389/fonc.2025.1725412

**Published:** 2025-12-15

**Authors:** Yu Zhang, Shirong Wu, Maolin Zhou, Hui Pan, Qing Fan, Jie Xie, Xue Xiao, Tian Zhang, Jinjun Shu, Yan Luo, Dongmei Ma, Qing Yang

**Affiliations:** 1 Nursing Department,Sichuan Cancer Hospital & Institute, Sichuan Cancer Center, School of Medicine, University of Electronic Science and Technology of China, Chengdu, China; 2 Department of Medical oncology,Sichuan Cancer Hospital & Institute, Sichuan Cancer Center, School of Medicine, University of Electronic Science and Technology of China, Chengdu, China; 3 Department of Anesthesiology, Sichuan Cancer Hospital & Institute, Sichuan Cancer Center, School of Medicine, University of Electronic Science and Technology of China, Chengdu, China

**Keywords:** machine learning, neuropathic pain, oncology, predictive model, surgery, thoracic

## Abstract

**Objectives:**

Neuropathic pain (NP) is a common and challenging complication following thoracic oncology surgery, characterized by complex etiological factors. However, effective predictive models for identifying high-risk patients are currently lacking. This study aims to determine the incidence and key risk factors associated with neuropathic pain following thoracic oncology surgery, and to construct and validate a series of machine learning-based risk prediction models, providing a scientific foundation for clinical decision-making.

**Methods:**

This study involved 647 patients who underwent thoracic oncology surgery at a specialized cancer hospital in Sichuan Province, China (November 2022 to December 2023). An information survey was designed to collect general demographic data and influencing factors. Outcome indicators were assessed using the Numeric Rating Scale (NRS) for postoperative acute pain and the Douleur Neuropathique 4 Questionnaire (DN4) for neuropathic pain evaluation. Using stratified sampling, the patients were divided into training (80%) and testing (20%) datasets. Univariate analysis and LASSO regression were employed to identify independent risk factors for postoperative neuropathic pain, resulting in the selection of seven factors for model inclusion. Subsequently, six machine learning models were developed using Python 3.11: logistic regression (LR), K-nearest neighbors (K-NN), random forest (RF), support vector machine (SVM), XGBoost, and LightGBM (LGBM). To enhance model accuracy, parameter tuning and ten-fold cross-validation were employed, and performance was evaluated using the testing set with the Area Under the Curve (AUC) metric. A visualization analysis of the model’s variable features was conducted, and the Shapley Additive Explanations (SHAP) values of the predictive models were calculated to identify the significant influencing factors and their respective impact levels on postoperative neuropathic pain in thoracic oncology.

**Results:**

The incidence of postoperative NP was 24.26%. The random forest model demonstrated the highest predictive performance (AUC = 0.86). SHAP value analysis revealed that the primary determinants for the onset of neuropathic pain include the surgical approach, the surgeon’s expertise, the quantity of thoracic drainage tubes, the duration of thoracic drainage tube placement, postoperative acute pain, and C-reactive protein (CRP).

**Conclusions:**

The random forest model effectively predicts neuropathic pain following thoracic oncology surgery, facilitating early screening and targeted interventions to improve outcomes.

## 1 Introduction

Neuropathic pain, a severe and debilitating disease affecting the peripheral and central nervous systems, is defined as “pain caused by lesions or diseases of the somatosensory system” (1, 2). This type of pain differs from common pain in several ways. Neuropathic pain encompasses both spontaneous and evoked symptoms, with the location of the pain typically corresponding to the area of damage. It is characterized by spontaneous pain, evoked pain, hyperalgesia, paresthesia, pinprick-like sensations, and electric shock-like features (3–5).

Surgical interventions are pivotal in addressing thoracic organ ailments, yet persistent postsurgical pain (PPSP) emerges as a prevalent complication across numerous surgical procedures (6). Notably, thoracic surgery is characterized by heightened levels of postoperative discomfort (7). Neuropathy-induced by nerve injury, termed neuropathic pain (NeuP), has consistently been pinpointed as the predominant etiology of PPSP. Empirical evidence indicates that the incidence of probable or definitive NeuP among patients experiencing prolonged pain following thoracic and breast surgeries stands at 66% and 68%, respectively (8).

Literature research indicates that the risk factors for neuropathic pain after thoracic oncology surgery primarily include age (9, 10), gender (11, 12), preoperative use of hypnotics (13), surgical method (14, 15), prolonged surgical duration (8), moderate to severe acute postoperative pain (16, 17), intraoperative blood loss, and postoperative drainage time (18). Currently, domestic studies on predictive models for postoperative pain in thoracic surgery mainly focus on the construction of models for chronic pain after thoracic surgery (19) and the risk factor analysis for neuropathic pain following thoracic oncology surgery (18), with most models being based on logistic regression methods. Recently, Lamin Juwara (20) and others have utilized machine learning techniques to develop a predictive model for neuropathic pain after breast cancer surgery, providing valuable references for this field.

Neuropathic pain, prevalent in thoracic oncology surgery, significantly impairs patients’ quality of life. The management of this pain hinges on its prediction and prevention. Numerous studies, both domestically and internationally, have sought to construct predictive models for neuropathic pain following thoracic oncology surgery. Most of these models employ traditional logistic regression (19, 21, 22). However, there is a notable lack of emphasis on postoperative neuropathic pain (18). Consequently, it is imperative to thoroughly investigate the risk factors for this type of pain in neuropathic pain following thoracic oncology surgery. By leveraging machine learning techniques, we aim to develop a predictive model for postoperative neuropathic pain, offering insights into its prevention and treatment.

## 2 Materials and methods

### 2.1 Data collection

The data for this study were obtained from patients who underwent thoracic oncological surgery at a tertiary hospital in Sichuan Province, China, between November 2022 and December 2023. This study received ethical approval from the Ethics Committee of a tertiary hospital in Sichuan Province, with the approval number SCCHEC-02-2022-160. All participants provided informed consent prior to their inclusion in the study.

Inclusion Criteria: Patients aged 18 years or older who were hospitalized and required elective thoracotomy via a standard posterolateral incision or video-assisted thoracoscopic surgery (VATS) for conditions including esophageal, pulmonary, or mediastinal tumors; provided informed consent and participated voluntarily; and underwent general anesthesia.

Exclusion Criteria: History of thoracic surgery;Patients who are already suspected of having neuropathic pain prior to surgery.

### 2.2 Anaesthetic and Surgical management

Anaesthesia was induced via intravenous administration of propofol (1.5–2.5 mg/kg), sufentanil (0.3 μg/kg), and cisatracurium (0.2 mg/kg). Anaesthesia was maintained with a continuous intravenous infusion of remifentanil (0.10–0.15 μg·kg⁻¹·min⁻¹) and inhalation of sevoflurane at a concentration of 1.5%–2.0%, adjusted to maintain a bispectral index (BIS) between 40 and 60. The remifentanil infusion rate was titrated according to intraoperative haemodynamic responses to maintain a heart rate of 60–80 beats per minute and to ensure blood pressure fluctuations remained within 20% of baseline values. Vasoactive agents were administered as necessary.

All surgical interventions were performed for oncological indications by a specialized thoracic surgery team. The surgical procedures were characterized and analyzed based on two key dimensions: Resected Organ and Anatomical Site in Thoracic Oncology Surgery: (a)Pulmonary Tumor Resections: Lobectomy, wedge resection, segmentectomy, and pneumonectomy. (b)Esophageal Tumor Resections: Included open McKeown, open Ivor-Lewis, totally minimally invasive McKeown, totally minimally invasive Ivor-Lewis, thoracoscopic-assisted McKeown with laparotomy, and thoracoscopic-assisted Ivor-Lewis with laparotomy. (c)Mediastinal Tumor Resections: Comprised open mediastinal lesion resection via lateral thoracotomy, video-assisted thoracoscopic surgery (VATS) for mediastinal lesion resection, and subxiphoid mediastinal lesion resection; Surgical Approach:(a)Uniportal Video-Assisted Thoracoscopic Surgery (VATS): Procedures performed through a single small incision.(b)Multiportal Video-Assisted Thoracoscopic Surgery (VATS): Procedures performed through multiple small incisions. (c)Open Thoracotomy: Procedures conducted via a standard posterolateral thoracotomy incision with rib retraction.

Drawing upon pertinent domestic and international literature, we have summarized the risk factors for neuropathic pain following thoracic oncology surgery. These were categorized based on preoperative, intraoperative, and postoperative stages, utilizing a self-compiled survey form. The specific survey items and corresponding time points are detailed in the **Table 1**.

**Table 1:** Survey items and corresponding time points.

Data collection time point	Collect content	Collection method
One day before the surgery	General demographic data	Medical record system access
	pain history, and related risk factors such as hypnotic drugs	On-site interview
The day of surgery	Intraoperative related risk factors	Medical record system access
1 and 3 days after the surgery	Postoperative pain level (NRS score)	On-site interview
	DN4 scale assessment	On-site interview
	Removal time of thoracic drainage tube	Medical record system access
7 days, 1 month, and 3 months after surgery	Postoperative Pain Level (NRS Score)	Telephone interview
	I-DN4 Scale Assessment	Telephone interview

### 2.3 Outcome

The evaluation of postoperative acute pain was facilitated through the Numeric Rating Scale (NRS), whereas the assessment of postoperative neuropathic pain was conducted utilizing the Douleur Neuropathique 4 questions (DN4) scale.

The Numeric Rating Scale (NRS) is a unidimensional tool that requires patients to rate their pain on a scale from 0 to 10, where 0 indicates no pain and 10 represents the most severe pain. In this study, an NRS score of ≥ 4 is considered indicative of postoperative acute pain.

The Douleur Neuropathique 4 questions (DN4) scale consists of 10 items, covering 7 symptoms (burning pain, freezing pain, electric shock-like pain, tingling sensation, stabbing sensation, numbness, and itching) and 3 physical examination components (reduced touch sensation, reduced pain sensation, and evoked pain). Each item is scored 1 point for a “yes” response. A total score of ≥ 4 points can diagnose neuropathic pain.

The abbreviated DN4 scale (I-DN4) primarily includes the aforementioned 7 symptoms. A score of ≥ 3 points on this scale is used to diagnose neuropathic pain.

### 2.4 Risk factors screening

During the data analysis phase, this study employed univariate analysis to identify factors influencing perioperative neuropathic pain in patients with thoracic oncology. Specifically, categorical variables (such as gender and marital status) were analyzed using the Pearson chi-square test; normally distributed numerical variables were assessed using the t-test; and non-normally distributed numerical variables were evaluated with the Mann-Whitney U test. To encompass potentially significant variables and avoid omitting important factors, variables with a p-value < 0.2 in the univariate analysis were considered statistically significant. This approach is supported by relevant academic papers, master’s theses, and professional textbooks (23–25), which commonly suggest that using a more lenient p-value (e.g., p < 0.2) in preliminary variable selection can help identify variables that may be overlooked with stricter thresholds, thereby enhancing the accuracy and robustness of subsequent models.

To further prevent variable overfitting, this study incorporated variables with a p-value < 0.2 from the univariate analysis into a LASSO regression model for secondary screening. The optimal model was determined based on cross-validation, ultimately identifying the core predictive factors for neuropathic pain following thoracic oncology surgery.

### 2.5 Model selection

Prior to model construction, a stratified random sampling technique was employed to segregate neuropathic pain following thoracic oncology surgery patients into training and testing datasets at an 8:2 ratio. The model’s development commenced with the training set, wherein parameter adjustments were made using grid search and random search techniques to ascertain optimal parameters (26). Additionally, ten-fold cross-validation within the training set was implemented to enhance its accuracy. Following this, patients from the test set were utilized for evaluation to assess the model’s performance. This assessment encompassed binary confusion matrix diagrams and various performance indicators, including accuracy, precision, recall, F1 score, sensitivity, specificity, and the area under the ROC curve (AUC). To conclude, the SHAP (27, 28) was employed to evaluate the contribution of each feature to the predictive model. The machine learning models adopted in this study comprised six types: logistic regression, nearest neighbor classification algorithm, random forest, support vector machine, extreme gradient boosting, and light gradient boosting.

### 2.6 Statistical analysis

Sample size calculation was conducted using a specialized formula proposed by Riley et al. (29) for predictive model research, following four key steps: estimating the sample size required to assess the overall outcome risk;calculating the sample size necessary to minimize model prediction error;evaluating the sample size needed to reduce model overfitting;considering the requirements for model optimization.Based on these steps, the minimum sample size determined was 668 cases, and the final study included 670 patients.

In the statistical analysis of fundamental information, which includes general demographic data and relevant preoperative and intraoperative indicators, we utilized IBM SPSS Statistics (version 26.0) for processing. Categorical data are presented as frequency, proportion, or percentage, while continuous data are expressed as mean ± standard deviation. To address missing data, we employed the MICE package for multiple imputation of longitudinal data. Sensitivity analysis revealed that even with a 30% reduction in sample size, the primary study results remained stable, indicating that the conclusions of this research demonstrate good robustness against various methods of handling missing data.

Utilizing Python 3.11, in conjunction with libraries such as numpy, matplotlib, openpyxl, packaging, pandas, pillow, Python-dateutil, scikit-learn, and scipy, we constructed a risk prediction model for neuropathic pain following thoracic oncology surgery. The efficacy of this model was assessed using metrics including accuracy, precision, recall, F1 score, sensitivity, specificity, and the area under the ROC curve. These evaluations facilitated an analysis of the significant features within the model’s variables.

## 3 Results

### 3.1 Basic information

This study ultimately comprised 647 neuropathic pain following thoracic oncology surgery patients, who were randomly segregated into a training set (517 cases) and a test set (130 cases), in an 8:2 ratio. The general demographic data for both groups are presented in **Table 2**. It can be inferred that there is no statistically significant difference in the distribution of age, gender, marital status, BMI, education level, ethnicity, and occupation between the training and test sets (*P*>0.05).

**Table 2:** Basic information.

Characteristics	Training set(n=517)	Test set (n=130)	*P*
Age			0.628
≤30	13(2.5%)	4(3.1%)	
30-60	302(58.4%)	81(62.3%)	
≥60	202(39.1%)	45(34.6%)	
Gender			0.492
Male	220(42.6%)	51(39.2%)	
Female	297(57.4%)	79(60.8%)	
Marital			0.819
Married	503(97.3%)	126(96.9%)	
Unmarried	14(2.7%)	4(3.1%)	
Nation			0.174
Han nationality	510(98.6%)	126(96.9%)	
Other minorities	7(1.4%)	4(3.1%)	
Education			0.089
Primary school and below	143(27.7%)	39(30%)	
Junior middle school	182(35.2%)	44(33.8%)	
High school	82(15.9%)	22(16.9%)	
College and above	110(21.3%)	25(19.2%)	
Occupation			0.207
Unemployed	322(62.3%)	76(58.5%)	
physical occupation	106(20.5%)	34(26.2%)	
Brain occupation	89(17.2%)	20(15.4%)	
BMI			0.375
≤18.5	22(4.3%)	8(6.2%)	
18.5-24.9	274(53%)	61(46.9%)	
≥25	221(42.7%)	61(46.9%)	
Smoking history			0.546
Yes	149(28.8%)	34(26.2%)	
No	368(71.2%)	96(73.8%)	
Drinking history			0.419
Yes	99(19.1%)	29(22.3%)	
No	418(80.9%)	101(77.7%)	

Categorical data are described as frequency (percentage) and were compared using the chi-square test. Whereas, the Mann-Whitney U test was employed for comparing the ordinal categorical variables (Education and Occupation) between groups.

### 3.2 Risk factors screening

**Figure 1:**
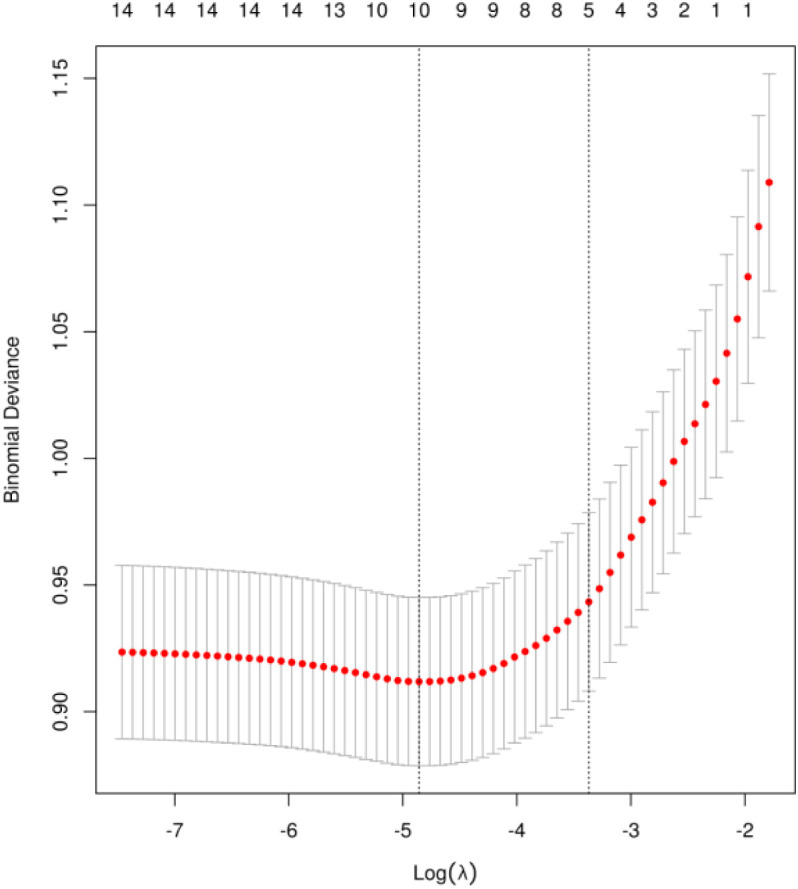
Risk factors screening using LASSO.

The univariate analysis results revealed that a total of 16 variables were associated with the manifestation of neuropathic pain following thoracic oncology surgery, including: education level, preoperative radiotherapy, preoperative chemotherapy, duration of preoperative persistent pain, surgical technique, surgical resection site, single-port and multi-port, surgeon, acute postoperative pain, postoperative wound infection, operation duration, The quantity of thoracic drainage tubes, Duration of thoracic drainage tube placement in days, and intraoperative blood loss, CRP, Postoperative analgesia methods, The comprehensive findings are presented in **Table 3**. To mitigate feature overfitting, this research incorporated statistically significant variables from the univariate analysis into Lasso regression for further refinement. The optimal model parameters were determined using cross-validation as depicted in **Figure 1**. Seven predictive variables exerting the most pronounced influence on neuropathic pain following thoracic oncology surgery: surgical resection site, single-port and multi-port, surgeon, acute postoperative pain, The quantity of thoracic drainage tubes, Duration of thoracic drainage tube placement in days and CRP.

**Table 3:** The factors under consideration are evenly distributed across both the training and test sets.

Factors	Non-neuropathic pain group	Neuropathic pain group	Statistical value	*P*
Age(Mean ± SD, Y)	56.41 ± 11.27	56.04 ± 10.87	*t* = 0.359	0.720
Gender			χ^2^ = 0.08	0.778
Marital status			χ^2^ = 0.234	0.628
Nation			χ^2^ = 1.402	0.236
Education			χ**^2^ = 4.51**	0.200*
Occupation			χ^2^ = 2.31	0.314
BMI(Mean ± SD)	23.35 ± 2.77	23.18 ± 3.08	*t*=-0.58	0.559
Smoking history			χ^2^ = 0.15	0.904
Drinking history			χ^2^ = 0.49	0.481
hypnotic drugs before surgery			χ^2^ = 0.70	0.400
long-term hypnotic drugs before surgery			χ^2^ = 0.11	0.738
Surgical history			χ^2^ = 0.859	0.354
Preoperative radiotherapy			χ**^2^ = 1.56**	0.200*
Preoperative chemotherapy			χ**^2^ = 2.63**	0.105*
Pain before operation			χ^2^ = 0.056	0.814
Duration of pain			χ**^2^ = 6.6**	0.159*
ASA			χ^2^ = 1.02	0.599
Operative method			χ**^2^ = 3.50**	0.173*
Surgical site			χ^2^ = 2.42	0.298
Surgical excision site			χ**^2^ = 22.87**	0.001*
Single-port and multi-port			χ**^2^ = 107.78**	<0.001*
Surgeon			χ**^2^ = 109.2**	<0.001*
Postoperative diagnosis			χ^2^ = 1.54	0.215
Postoperative acute pain			χ**^2^ = 24.06**	<0.001*
Wound infected			χ**^2^ = 11.53**	0.001*
Operation Time(M(Range), min)	**89.43 (55**-115)	**98.40 (60**-130)	**Z=-2.349**	0.019*
The quantity of thoracic drainage tubes			χ**^2^ = 4.24**	0.121*
Duration of thoracic drainage tube placement in days(M(Range), d)	**3.43(2-4)**	**4.18(2-5)**	**Z=-1.57**	0.115*
Intraoperative blood loss(M(Range), ml)	**82.8(30-100)**	**109.17(50-100)**	**Z=-2.34**	0.003*
Diabetes			χ^2^ = 0.390	0.520
CRP			χ**^2^ = 7.202**	0.007*
Postoperative analgesia methods			χ**^2^ = 1.96**	0.160*

Operative method: Surgeries are classified into three categories based on the surgical approach: thoracoscopic surgery, open surgery, and surgeries that transition from thoracoscopic to open procedures.

Surgical Site: This refers to the primary organ sites that undergo surgery, including the lungs, esophagus, and mediastinum.

Surgical excision site: For lung surgeries, the classification is based on lung lobes and specific surgical techniques. This includes: left upper lobe (resection/lobectomy/wedge resection), left lower lobe (resection/lobectomy/wedge resection), right upper lobe (resection/lobectomy/wedge resection), right middle lobe (resection/lobectomy/wedge resection), and right lower lobe (resection/lobectomy/wedge resection). Non-lung surgeries are recorded directly as mediastinal surgeries or esophageal surgeries.

Surgeon: The classification is based on the total number of thoracic surgeries performed by the primary surgeon in the year prior to this study. The classification is as follows: (High-level surgeon: annual surgical volume ≥ 2000 cases; Mid-level surgeon: annual surgical volume between 1000–2000 cases; Low-level surgeon: annual surgical volume < 1000 cases).

Bold and * values indicate variables with P < 0.2 in the univariate analysis, which were subsequently included in the LASSO regression for further selection.

### 3.3 Postoperative acute pain and neuropathic pain

The assessment of postoperative acute pain employs the NRS score, with acute pain defined as an NRS score of ≥4 at postoperative intervals T1 (one day post-surgery), T2 (three days post-surgery), and T3 (seven days post-surgery). At T1, 120 cases (18.54%) reported experiencing postoperative acute pain, 70 cases (10.81%) at T2, and 36 cases (5.56%) at T3. The incidence of postoperative acute pain peaked on the first day post-surgery, exhibiting a progressively decreasing trend on the third and seventh days post-surgery. When accounting for unique patients only in T1, T2, and T3 combined, a total of 140 distinct patients experienced acute pain within seven days following thoracic surgery, resulting in an overall incidence rate of postoperative acute pain of 21.63%.

The evaluation of neuropathic pain following thoracic oncology surgery was performed using the DN4 and I-DN4 scales. The specific incidence rates were as follows: 2.93% at one day post-operation; 3.4% at three days post-operation; 4.94% at seven days post-operation; 12.51% at one month post-operation; and 8.5% at three months post-operation. The peak incidence of postoperative neuropathic pain occurred at one month post-operation. Out of a total of 157 patients who experienced postoperative neuropathic pain, 56 had previously encountered acute postoperative pain. The incidence of postoperative neuropathic pain was found to be 24.26%.

### 3.4 Model test result

**Figure 2:**
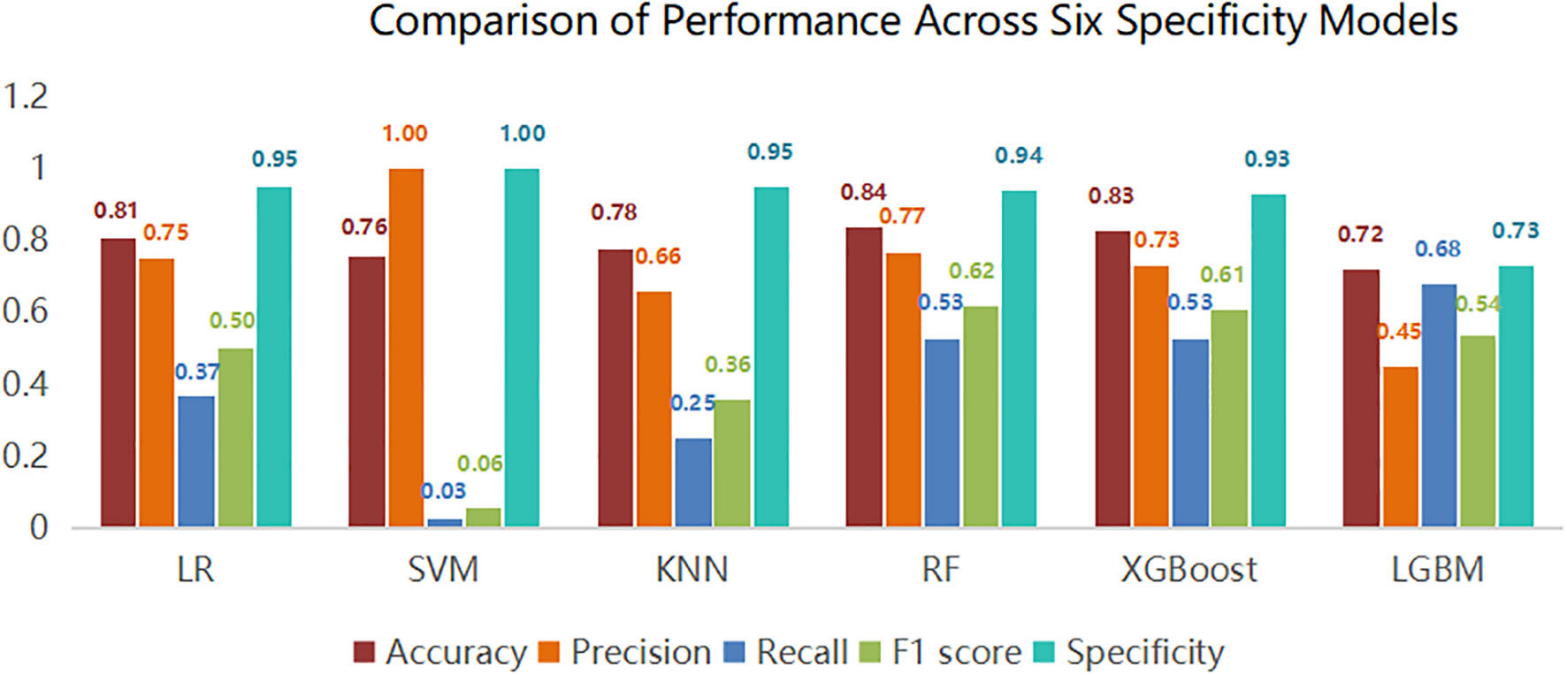
Comparison of performance metrics for models on the test set.

**Figure 3:**
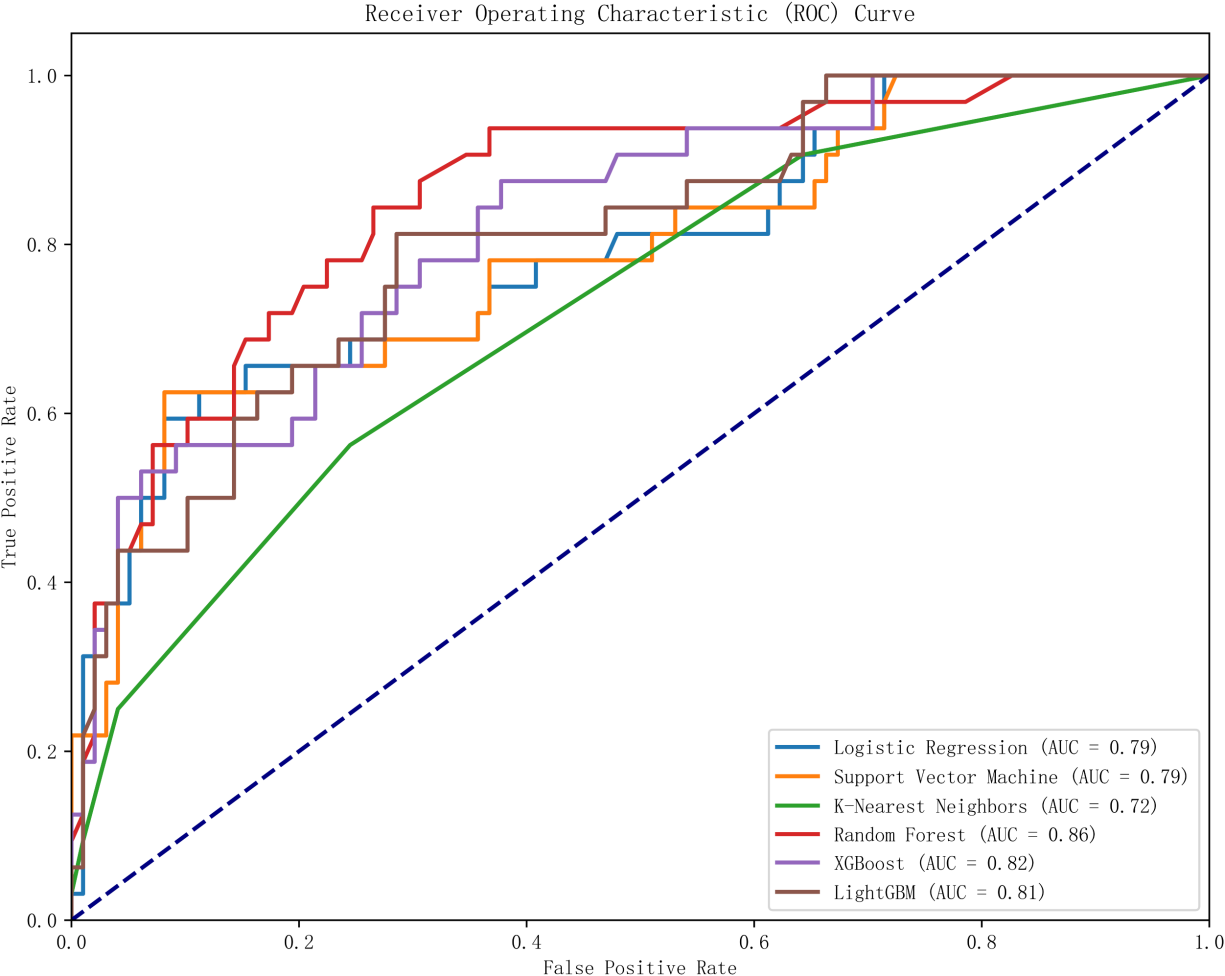
The ROC curve of six models on the test set.

**Figure 4:**
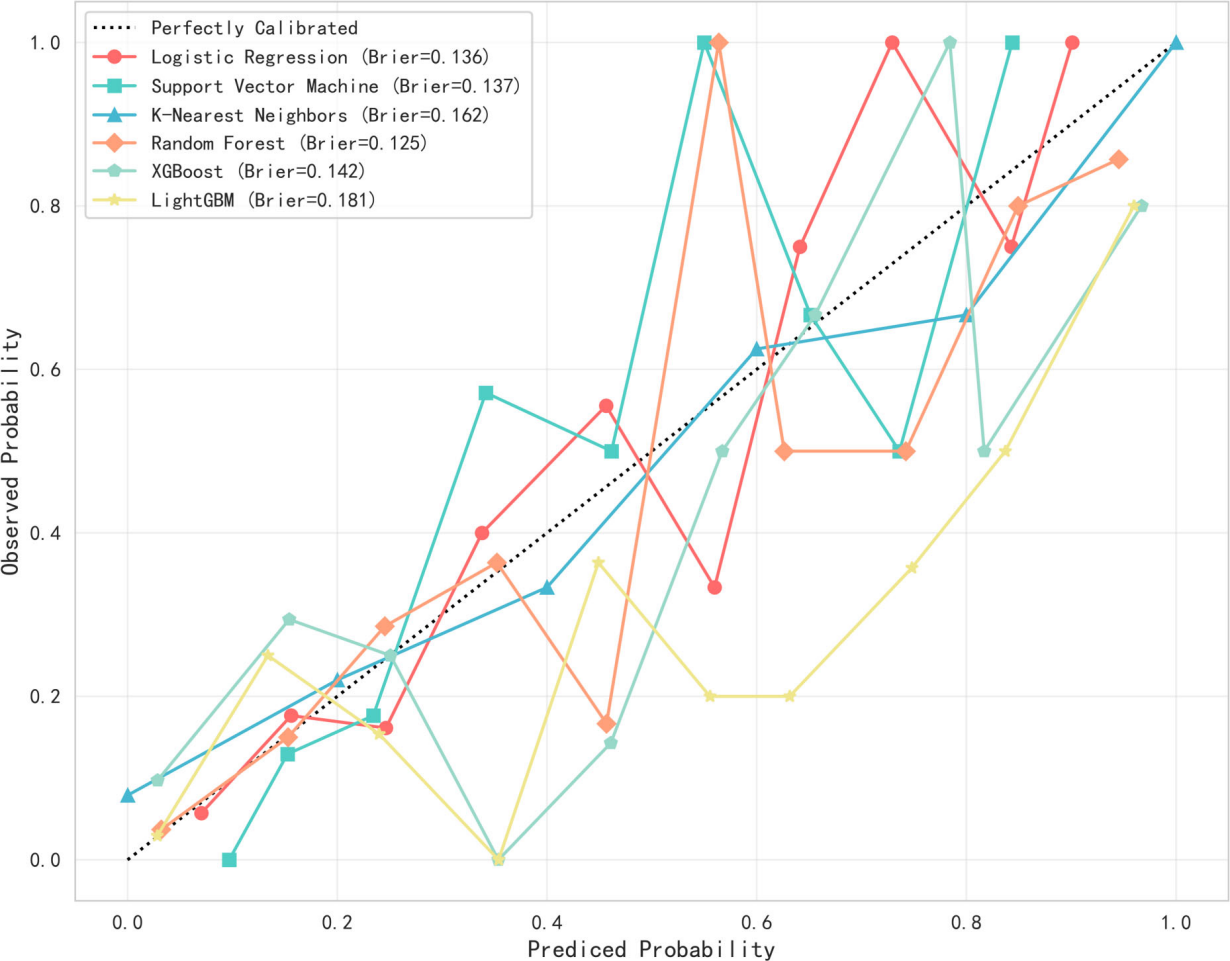
The calibration curves of six models. Probability calibration curves for six machine learning models. The x-axis represents the predicted probabilities from the models, while the y-axis shows the actual observed probabilities. The diagonal line indicates the reference line for perfect calibration. The Brier score (with lower values indicating better performance) reveals that the random forest model (0.125) exhibits the best calibration performance.

This study utilized Python software to construct six distinct machine learning models, subsequently comparing their evaluation metrics on a test set following ten-fold cross-validation. In terms of accuracy, the results from the Random Forest (RF), XGBoost, and Logistic Regression (LR) models were found to be comparable. These models demonstrated superior accuracy in predicting both true negatives (non-neuralgia) and true positives (neuralgia) compared to the other three models. In terms of Area Under the Curve (AUC) values, the RF model surpassed the other five prediction models.Ten-fold cross-validation was used within the training set to enhance its accuracy. The average ROC-AUC and 95% CI were as follows: LR at 0.79 (95% CI 0.74-0.80), SVM at 0.79 (95% CI 0.73-0.78), KNN at 0.72 (95% CI 0.70-0.75), RF at 0.86 (95% CI 0.71-0.80), XGBoost at 0.82 (95% CI 0.69-0.80), and LGBM at 0.81 (95% CI 0.71-0.80). The result is shown in **Figure 2**. In internal cross-validation, both the RF and XGboost models yielded superior average roc_auc results compared to other models.The result is shown in **Figure 3**. To better demonstrate the calibration degree of different models, the calibration curves are also shown in **Figure 4**.

**Figure 5:**
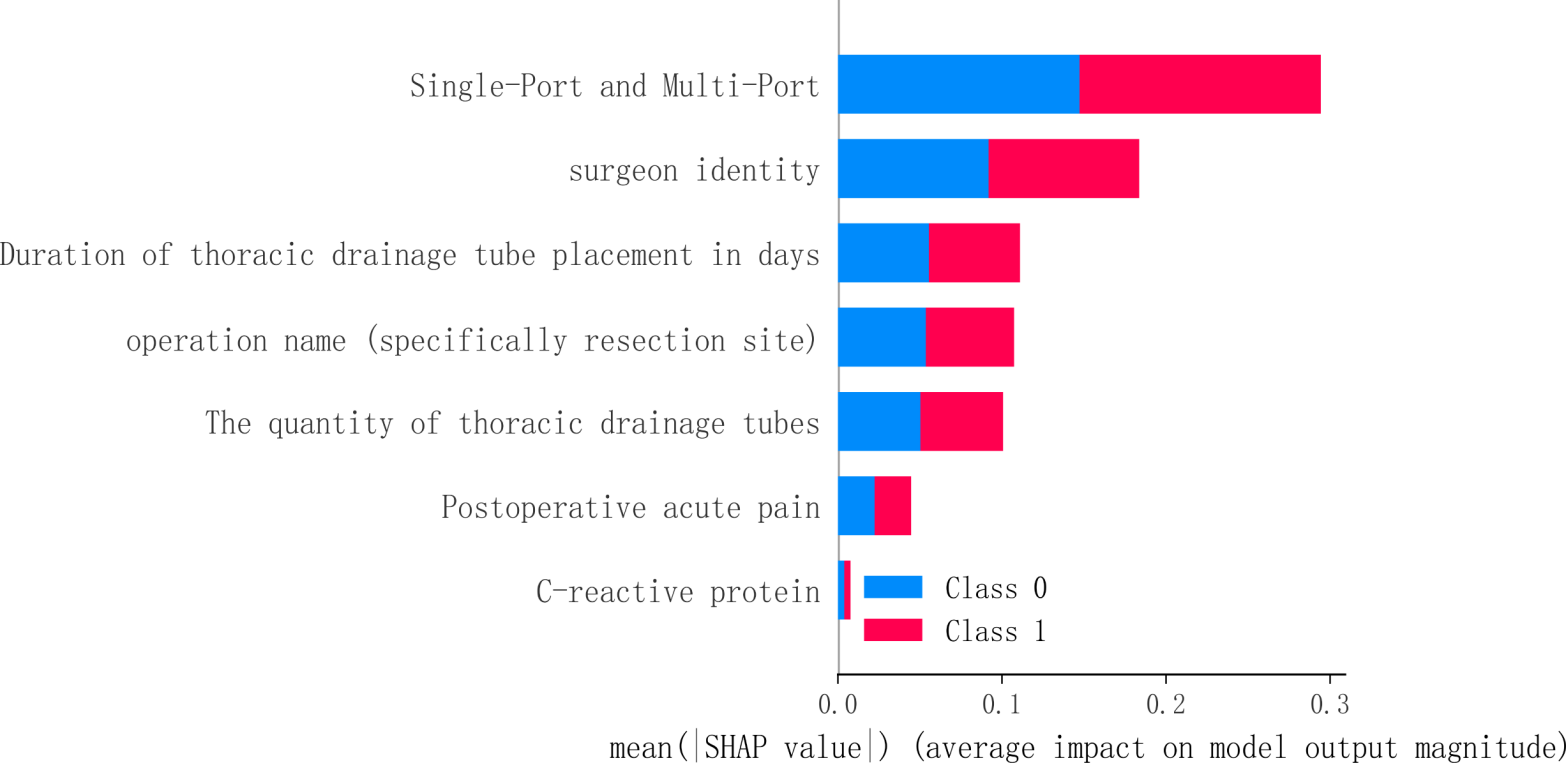
The SHAP value of the random forest in feature influence. This figure utilizes SHAP values to interpret the model, revealing key predictive factors and their impact. Features located higher up (such as the surgical approach) have a greater average influence on the model’s output. The horizontal position and color of the points together illustrate how feature values affect predictions: red points indicate that the feature value pushes the prediction towards the occurrence of neuropathic pain (Class 1), while blue points suggest a push towards the absence of neuropathic pain (Class 0).

To identify the most influential features in the model predictions, we performed a visual analysis using SHAP (SHapley Additive exPlanations) values. This analysis was applied to the Random Forest (RF) model, revealing the following key features, ranked by their mean absolute SHAP value: Single-port and multi-port (0.147), surgeon identity (0.092), duration of thoracic drainage tube placement in days (0.055), operation name (0.053), the quantity of thoracic drainage tubes (0.050), postoperative acute pain (0.022), and CRP (0.004). The results are visualized in **Figure 5**.

## 4 Discussion

### 4.1 Analysis of neuropathic pain conditions

Intercostal nerve injury is identified as a significant pathogenic factor contributing to neuropathic pain following thoracic surgery, with reported incidence rates ranging from 23% to 66% (8, 30). This study determined that the incidence rate of neuropathic pain thoracic oncology was 24.26%. The peak incidence of postoperative neuropathic pain occurred at one month post-operation. International researchers have conducted evaluations and follow-ups on intercostal nerve injury in patients undergoing thoracic oncological surgery (31). They measured the current perception threshold of the intercostal nerve before surgery and at intervals of one, two, four, twelve, and twenty-four weeks after surgery. The findings indicated that the current perception threshold significantly increased at 2000 Hz one week post-operation, and the intensity of persistent pain was more pronounced at four and twelve weeks post-operation. These results align with the timing of the occurrence of postoperative neuropathic pain observed in this study.

### 4.2 Analysis of risk factors associated with postoperative neuropathic pain

#### 4.2.1 Single-port and multi-port

The relationship between single-port, multi-port, and neuropathic pain following thoracic oncology surgery is evident (32). This study demonstrated that multi-port and open surgery significantly increased the incidence of neuropathic pain following thoracic oncology surgery compared to single-port surgery. The single-port incision is performed within a wider intercostal space on the anterior axillary line, while multi-port is conducted within the narrower intercostal space on the posterior axillary and midaxillary lines. This results in thinner muscle layers, which are more likely to cause stress, disorder, and potential damage to the lateral intercostal nerve. Furthermore, Benedetti et al. found that anterior thoracotomy, compared to the posterolateral approach, is less likely to cause nerve injury and subsequent chronic pain (33). Therefore, for multi-port thoracic surgery, medical staff should prioritize monitoring patients’ postoperative pain and facilitating their recovery.

#### 4.2.2 Surgeon

This study identifies surgeons as a significant factor influencing the incidence of postoperative neuropathic pain, an area that currently remains under-researched. It is widely recognized that neuropathic pain following thoracoscopic surgery primarily results from continuous compression of the intercostal nerves by the thoracoscope during the procedure, as well as persistent stimulation caused by the postoperative chest drainage tube (34). Given that intercostal nerve injury is a key contributor to neuropathic pain, the surgical technique employed by the surgeon is particularly critical.

The study further demonstrates that the likelihood of patients developing postoperative neuropathic pain decreases as the annual surgical volume of the medical team increases—a correlation closely associated with the seniority and surgical experience of the surgeons. Surgeons with greater operative experience and higher case volumes are able to employ more refined dissection techniques, more precise electrocautery, and gentler tissue retraction, thereby significantly reducing tissue trauma, attenuating local inflammatory responses, and effectively alleviating postoperative pain.

On the other hand, the placement and selection of chest drain size also depend on the surgeon’s expertise. More experienced surgeons tend to shorten the duration of chest tube drainage. Studies have also shown that thin-bore chest tubes exert significantly less compression on the intercostal nerves, skin, and surrounding tissues compared to thick-bore tubes. Since postoperative pain largely stems from intercostal nerve compression, the use of thin-bore tubes can markedly reduce pain. In addition, incisions for thin-bore tubes do not require suture fixation, resulting in lower local tension and promoting better wound healing (35). Furthermore, experienced surgeons demonstrate more nuanced and effective approaches in formulating analgesic regimens and managing pain.

#### 4.2.3 The quantity and duration of chest drainage tubes

This study demonstrates that the quantity and duration of chest drainage tubes independently influence neuropathic pain following thoracic oncology surgery. This finding aligns with the research conducted by domestic scholar Cheng Shaoyi (18) and international researchers Miyazaki, Takuro et al. (36)The insertion of chest drainage tubes during thoracic surgery is a significant contributor to intercostal nerve damage. Miyazaki, Takuro and colleagues conducted a perception threshold test on patients with inserted chest drainage tubes to objectively evaluate any potential damage to the intercostal nerve. The results indicated that the impact of chest drainage tube placement on intercostal nerve function. The more drainage tubes are present or the longer their placement duration, the greater the likelihood of pulling and compressing the intercostal nerve, thereby increasing the risk of damage. Therefore, for patients with numerous chest drainage tubes and extended durations, if feasible, the chest drainage tube should be removed as soon as possible. Alternatively, a more flexible and softer drainage tube could be used, and the angle of the drainage tube adjusted in a timely manner. This may mitigate or prevent the occurrence of intercostal nerve damage and associated pain (37).

#### 4.2.4 Surgical resection sites

Currently, there is a paucity of studies that thoroughly explore the issue of neuropathic pain resulting from surgical resection sites in thoracic oncology surgery. The findings of this study indicate that surgical resection sites on the right side are more likely to induce neuropathic pain than those on the left side. To date, no literature has been identified that corroborates this result. We examine potential reasons for this discrepancy. Firstly, the prevalence of malignant tumors in the right lung exceeds that in the left lung. Secondly, existing literature suggests that the function of the right lung constitutes 55%-60% of total lung capacity, and surgeries involving the right lung are more likely to result in hypoxia (38). We propose that hypoxia may exert a certain influence on intercostal nerve damage.

#### 4.2.5 Postoperative acute pain

Postoperative acute pain is positively correlated with postoperative neuropathic pain (16). The etiology of postoperative acute pain may be attributed to intercostal nerve damage, inflammation of chest wall structures proximate to the incision, injury to lung parenchyma or pleura, and the placement of a chest drainage tube. Notably, intercostal nerve damage can result from nerve compression during rib retraction or contraction during suturing, or it might be induced by stimulation from the insertion of a chest drainage tube. This study further underscores that postoperative acute pain significantly influences postoperative neuropathic pain.

#### 4.2.6 C-reactive protein (CRP)

The findings suggest that CRP levels may contribute to postoperative neuropathic pain. As a sensitive marker of systemic inflammation, elevated CRP could indicate the inflammatory response following nerve tissue damage. However, a direct causal relationship between CRP and neuropathic pain remains unclear. It is uncertain whether CRP-mediated inflammation directly triggers pain or if secondary pain-induced inflammation raises CRP levels. These observations suggest that inflammation plays a key role in the pathophysiology of postoperative neuropathic pain. Standardized perioperative pain management is essential. Preoperatively, patients should be educated on pain scoring, and postoperative assessments should ensure accurate pain documentation, allowing for timely interventions and personalized care (39).

### 4.3 Pharmacological and Non-Pharmacological Pain Management

A range of pharmacological agents, including gabapentinoids, tricyclic antidepressants (TCAs), NMDA receptor antagonists (e.g., ketamine), and local anesthetics, have shown potential in preventing postoperative neuropathic pain. Gabapentinoids and TCAs are especially effective and considered first-line treatments (40, 41). Gabapentinoids suppress neuronal hyperexcitability by modulating calcium channels, while TCAs inhibit the reuptake of norepinephrine and serotonin. For localized pain, topical agents such as lidocaine or capsaicin patches may offer viable alternatives (42, 43). Non-pharmacological strategies like psychological support and physical therapy can complement pharmacological treatments, enhancing the overall effectiveness of pain management.

### 4.4 Machine Learning Models for Pain Prediction

Random Forest, as an ensemble learning method, enhances model accuracy and robustness by integrating the predictions of multiple decision trees, while also demonstrating strong resistance to overfitting. When dealing with high-dimensional data or small sample sizes, this model typically maintains good generalization performance. The results of this study indicate that the Random Forest model exhibits strong competitiveness across various evaluation metrics; it excels not only in discriminatory power (AUC) but also achieves a favorable balance between sensitivity and specificity, highlighting its clear advantages in classification tasks.

**Figure 6:**
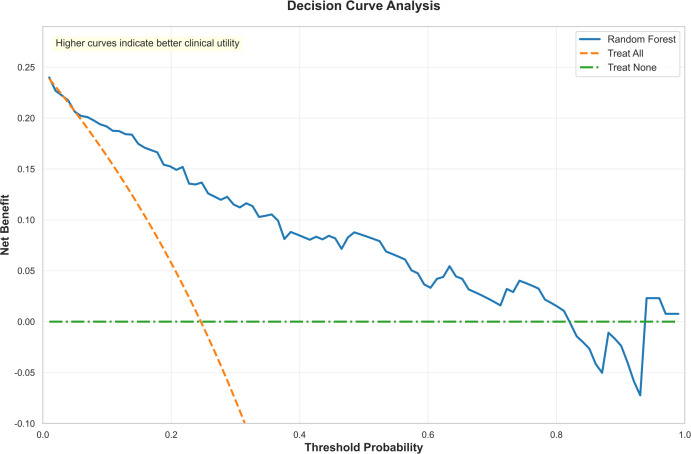
The decision curve analysis of the random forest. Decision curve analysis to evaluate the clinical utility of the random forest model. The y-axis represents the net benefit, while the x-axis indicates the threshold probability. The results demonstrate that across a wide range of threshold probabilities (approximately 0.1 to 0.7), the net benefit of the random forest model (orange curve) consistently exceeds that of the extreme strategies of “treat all” and “treat none.” This indicates that the model possesses substantial clinical utility and can assist physicians in making better-informed decisions in most scenarios.

Compared to traditional logistic regression models, the Random Forest model can also incorporate feature importance analysis and SHAP values, effectively revealing the decision-making mechanisms of the model. Additionally, we conducted decision curve analysis for the RF model, which demonstrates its substantial clinical utility and ability to assist physicians in making better-informed decisions in most scenarios, as illustrated in **Figure 6**.

### 4.5 Limitations

The limitations of this study include the fact that intraoperative anesthetic agents were not analyzed as independent influencing factors, primarily due to insufficient evidence in the existing literature explicitly identifying them as determinants. Nevertheless, we have thoroughly documented the anesthesia management protocols of the patients in the manuscript, providing foundational data for future in-depth research.

Additionally, the continuous inclusion of patients undergoing thoracic oncology surgery in this study, coupled with the widespread adoption of minimally invasive techniques, has resulted in a relatively limited number of open thoracic surgery cases being included. Existing studies suggest that open surgery may be one of the independent risk factors for neuropathic pain following thoracic oncology surgery. Due to the insufficient sample size of open surgeries in this study, we may not have been able to adequately assess the impact of this factor. In future research, increasing the sample size will facilitate a systematic comparison of the efficacy and complication risks between open and minimally invasive surgeries. Finally, the single-center design and the fact that data collection, model development, and validation all relied on the same source limit the generalizability of our findings. Multi-center studies incorporating more diverse populations are essential to enhance the model’s external validity.

## 5 Conclusions

The prevalence of neuropathic pain following thoracic oncology surgery is 24.2%, with the highest incidence occurring one month post-operation. Factors such as surgical approach, surgeon experience, duration of chest drainage tube placement, resection site, number of drainage tubes, acute postoperative pain,CRP are independent risk factors for neuropathic pain following thoracic oncology surgery. This study is unique in its population and methodology, as no predictive models for neuropathic pain following thoracic oncology surgery, despite models for other subspecialties like breast surgery. The RF machine learning model demonstrates superior performance in predicting neuropathic pain and offers a reliable tool for clinical use. It can aid in more accurate screening for neuropathic pain, inform prevention and intervention strategies, and raise awareness among patients about their risk for developing this condition.

## Data Availability

The original contributions presented in the study are included in the article/supplementary material. further inquiries can be directed to the corresponding author/s.
